# Lamalbid, Chlorogenic Acid, and Verbascoside as Tools for Standardization of *Lamium album* Flowers—Development and Validation of HPLC–DAD Method

**DOI:** 10.3390/molecules25071721

**Published:** 2020-04-09

**Authors:** Monika E. Czerwińska, Eleonora Kalinowska, Dominik Popowski, Agnieszka Bazylko

**Affiliations:** 1 Department of Pharmacognosy and Molecular Basis of Phytotherapy, Medical University of Warsaw, 1 Banach street, 02-097 Warsaw, Poland; dominik.popowski@wum.edu.pl (D.P.); agnieszka.bazylko@wum.edu.pl (A.B.); 2 Students’ Scientific Association at the Department of Pharmacognosy and Molecular Basis of Phytotherapy, Medical University of Warsaw, 1 Banach street, 02-097 Warsaw, Poland; eleonora_kalinowska@op.pl

**Keywords:** iridoids, phenylpropanoids, phenolic acids, HPLC–DAD, Lamiaceae

## Abstract

Preparations from the flowers or herb of the white dead nettle (*Lamium album* L.) are recommended for the treatment of upper respiratory tract disorders or as a topical medication for mild inflammation of the throat, mouth, and skin. Taking into consideration the significance of *L. album* in traditional medicines across Europe, as well as the lack of studies describing the quantities of their most abundant constituents, we aimed to design a high-performance liquid chromatography coupled with diode-array detection (HPLC–DAD) method for potential standardization procedures of extracts from flowers of *L. album*. The HPLC–DAD method was developed and validated for quantification of iridoids (lamalbid), phenolic acids/depsides (chlorogenic acid), phenylpropanoids (verbascoside), and flavonoids (rutin; quercetin malonylhexoside; tiliroside) in aqueous and ethanolic-aqueous extracts of *Lamii albi flos*. The method was specific, accurate, and precise. Lamalbid was the most abundant compound both in aqueous (39.09 ± 1.02 m/g dry weight) and ethanolic-aqueous (26.66 ± 0.64 m/g dry weight) extracts. The quantities of selected compounds, except for chlorogenic acid and tiliroside, were higher in the aqueous extract than in the ethanolic-aqueous one. In conclusion, the method developed allowed for quantitation of compounds from different classes. In particular, chlorogenic acid and verbascoside have been proposed as reference compounds for routine quantitative control of *Lamii albi* *flos*.

## 1. Introduction

Progress towards ensuring and promoting human health requires guarantees in the quality of both food and medicinal plant products. In the latter case, quality specifications are usually provided in the international pharmacopoeias, which however do not include a large number of herbal products. Nowadays, this particular case is widely discussed [1,2]. Nevertheless, the tests designed for the quality control of neglected plants are still required in the developing states of Europe [1]. Even though a wide range of techniques were developed for analysis of polyphenol profiles of herbs, these methods generally missed specificity and sensitivity [3]. Therefore, there is still a need to ensure the quality of traditionally used medicinal products by using modern control techniques and applying suitable standards [1]. It is important to discuss validation procedures, which include identification and quantitative analysis of active compounds, in addition to the control of impurities [4].

A large variety of Lamiaceae species are used as medicinal and aromatic plants all over the world. Some of these are listed among the most popular spices and herbs (e.g., basil, peppermint, thyme), while many others are used as herbal remedies (like purple giant hyssops used in traditional Chinese medicine) [5,6]. The pharmacopoeia monograph of *Marrubii herba*, also belonging to the family Lamiaceae, provides the HPLC-based determination of diterpenes, such as marrubin (Eur. Ph. 9th Ed.) [7]. In addition, many raw materials from the family Lamiaceae, such as *Menthae piperitae folium*, *Melissae folium*, *Thymi herba*, *Leonuri cardiacae herba,* or *Prunellae spica* are used as medicinal plants, standardized according to the monographs of the European Pharmacopoeia (Eur. Ph. 9th Ed.) [7]. *Leonuri cardiacae herba,* or *Prunellae spica*, as raw materials that are poor in essential oil, are standardized for other (non-volatile) compounds. *Leonuri cardiacae herba* is standardized for flavonoid content, whereas *Prunellae spica* is standardized for the content of triterpene compounds. However, plant materials or their preparations obtained from plants belonging to the subfamily Nepetoideae, rich in essential oils, are also more frequently standardized for non-volatile groups of chemical compounds. Examples are *Melissae folium* and *Menthae piperitae foli extractum siccum*, which are standardized for rosmarinic acid (depside) content.

The flowers of the white dead nettle have been used officially since the nineteenth century, when the dried and peeled corolla were included in the French Pharmacopoeia (10th Ed.) [8]. White dead nettle preparations have only been used traditionally in the treatment of kidney and bladder complaints to enhance renal excretion of water; while in folk medicine, they have long been used in vaginal infections to treat leucorrhoea or to arrest uterine hemorrhage, and to reduce excessive menorrhagia [9,10]. Recent studies have provided reports on the activity of extracts from *L. album* herb in vivo, such as a decrease of mRNA expression of liver cyclooxygenase-2 (COX-2), and a decrease of glycogen synthase-3 in diabetic rats, as well as the relaxation of rat tracheal [11,12,13]. The haemostatic properties of butanolic extract of *L. album* were investigated by haemostatic test-tail bleeding time determination and by acenocoumarol carrageenan test in rats [14].

Phenylpropanoids have been established as the most diverse class of compounds in the flowers and herb of *L. album*. Lamalboside, also named lamiuside A, has been recognized as the most characteristic phenylpropanoid ester of *L. album*, in addition to verbascoside (**6**) [15]. In our previous study others phenylpropanoids, such as lamiusides B, C, and E, as well as phlinoside D, have been found in this species for the first time. We established that this class of compounds was likely to determine the potential role of white dead nettle preparations in cytokines related inflammation [16,17]. However, iridoids such as lamalbid (lamiridoside, **1**), lamiol, and caryoptoside (**2**) seem to play a role of chemotaxonomic markers of species from the genus *Lamium* [18,19]. Last but not least, phenolic acids and/or depsides, in particular chlorogenic acid (**3**), as well as flavonoids have also been identified in the extracts of the flower and herb of *L. album* [17].

To date, the extracts tested in the biological models were standardized mainly based on total phenolic and flavonoids content expressed as gallic acid and quercetin equivalents, respectively [12,13,20]. The quantified phenolic compounds in the purified ethanolic extract of *L. album* accounted for 500.7 ± 50.0 mg g^−1^ of extract [21]. In some studies, the contents of phenolic acids were determined in the methanolic and chloroform extracts of *L. album* herb, and in the in situ and in vitro cultivated plants with HPLC–DAD method of external standards [22,23]. However, the quantification of phenolic acids, flavonoids, and quercetin glycosides was performed using different mobile phases in these studies [23]. To the best of our knowledge, there is no available literature providing data on the simultaneous quantitation of white dead nettle phytochemicals representing the different classes of compounds, such as lamalbid (**1**), chlorogenic acid (**3**), verbascoside (**6**), and quercetin malonylhexoside (**7**), as well as HPLC-based quantitative method for analysis of aqueous and ethanolic-aqueous extracts of *L. album.* Therefore, due to the traditional significance of *L. album* and the lack of the studies describing the quantities of their most abundant constituents in aqueous and ethanolic-aqueous extracts, we aimed to develop and validate HPLC–DAD method for potential standardization procedures of this plant material. We decided to focus on quantitation of iridoids and phenolic compounds. In particular, these latter compounds occur widely in natural products and can be easily used for standardization.

## 2. Results and Discussion

**Figure 1:**
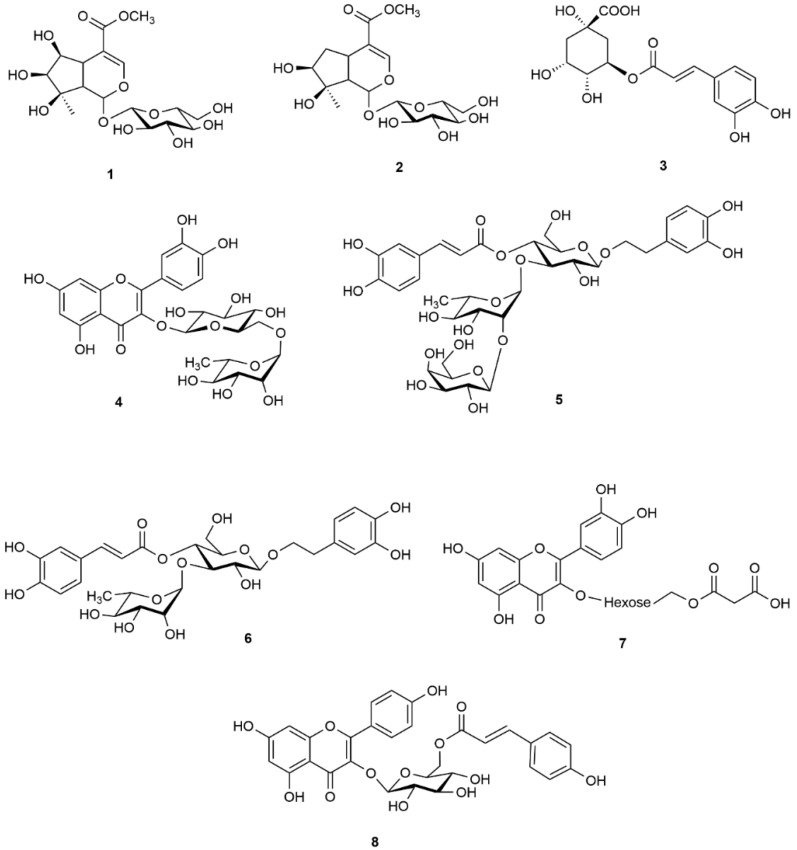
Structures of compounds selected for quantitation with high-performance liquid chromatography coupled with diode-array detection (HPLC–DAD) method.

The Lamiales is a wide order of plants among which well-known, or economically important members, play a role in traditional medicine or diet. In this study the standardization procedure of *Lamii albi flos* extracts was developed. Phenolic compounds, phenylpropanoids, and iridoids play a key role in the activity of extracts as well as being meaningful chemical tools, particularly chlorogenic acid (**3**), verbascoside (**6**), and tiliroside (**8**), which are useful in the quantitative analysis of plant materials (Figure 1).

**Figure 2:**
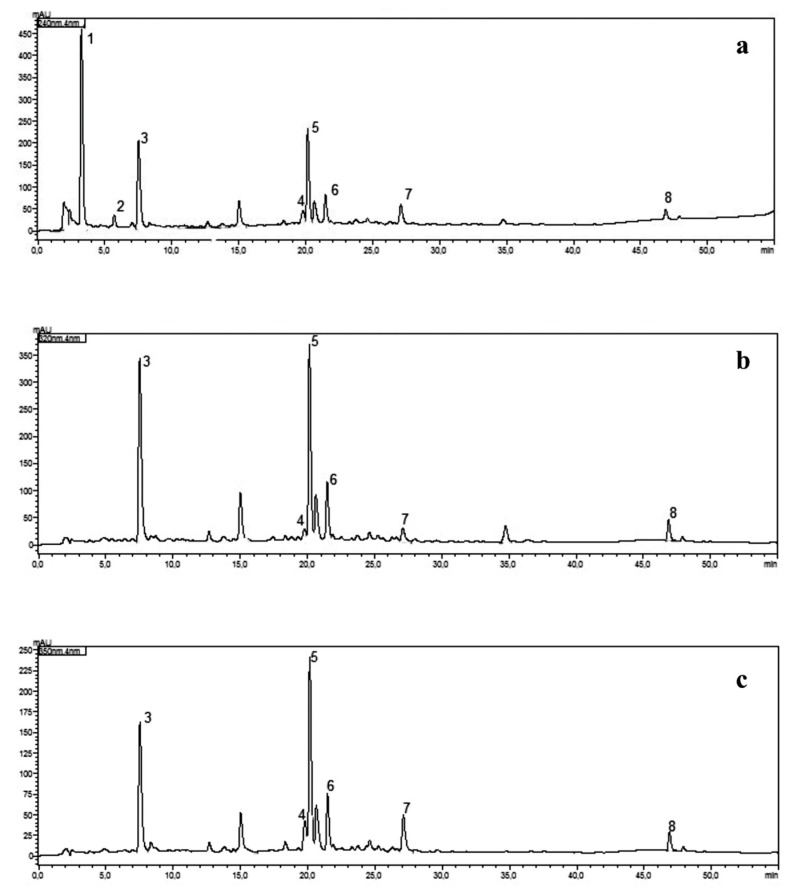
HPLC–DAD chromatograms of ethanolic-aqueous extract detected at: 240 nm (**a**), 320 nm (**b**), 350 nm (**c**); 1—lamalbid, 2—caryoptoside, 3—chlorogenic acid, 4—rutin, 5—lamiuside A, 6—verbascoside, 7—quercetin malonylhexoside, 8—tiliroside.

In order to optimise chromatographic conditions, various tests were performed. The assumptions of the developed method were to find a gradient that allows for good separation of chemical compounds with the shortest possible analysis time. The starting gradient, from which the search for a proper method of separation of chemical substances in extracts from *Lamium album* flower began, was a gradient, 0–60 min, 5–60% B. Using the above-mentioned gradient, eight chemical substances used as standards were detected in the tested extracts. However, the retention time of the first detected compound (lamalbid) was relatively long (approx. 20 min). In addition, the separation of rutin (**4**) and lamiuside A (**5**) was not sufficient for quantification. To reduce the retention time of individual chemical compounds, the above gradient has undergone several modifications. The biggest problem while developing the method was a lack of separation of the pairs of peaks: lamalbid (**1**)/chlorogenic acid (**3**) and rutin (**4**)/lamiuside A (**5**). In order to achieve satisfactory separation and analysis time, it was decided to introduce a multi-stage gradient. Several analysis variants were tested, in which initial and final concentrations of phase B, and an increase of the phase B concentration over time at each stage of the analysis, were modified. This made it possible to obtain shorter retention times for the tested compounds, satisfactory separation of overlapping peaks, and a shortening the total analysis time by 10 min. Finally, a three-step gradient solvent system, 0–17 min, 10–19% B, 17–37 min, 19–21% B, and 37–50 min, 21–40% B, was used. Thanks to the above method, it was possible to separate and identify eight tested chemical substances present in the aqueous and ethanolic-aqueous extracts of white dead nettle flowers. Retention times for: lamalbid (**1**), caryoptoside (**2**), chlorogenic acid (**3**), rutin (**4**), lamiuside A (**5**), verbascoside (**6**), quercetin malonylhexoside (**7**), and tiliroside (**8**), were 3.26, 5.70, 7.51, 19.77, 20.05, 21.38, 27.07, and 46.86 min, respectively. Chromatograms of ethanolic-aqueous extract detected at: 240 nm, 320 nm, and 350 nm, obtained using the developed method are shown in Figure 2.

Due to the limited amount of standards of caryoptoside (**2**) and lamiuside A (**5**), the method was validated for the quantitative determination of six compounds: lamalbid (**1**), chlorogenic acid (**3**), rutin (**4**), verbascoside (**6**), quercetin malonylhexoside (**7**), and tiliroside (**8**) (Supplementary Materials, Figures S1–S6). The parameters of validation of the tested compounds are presented in Table 1.

In both tested extracts, the most abundant compounds were iridoids such as lamalbid (**1**). There is evidence of high variability of iridoid and phenolic compounds occurrence and concentration depending on both environmental factors, such as light exposure or irrigation, and extraction parameters (solvent, plant material:solvent ratio, temperature, and time of extraction) [24,25,26,27]. The influence of plant material:solvent ratio and temperature as well as their impact on the total phenol response was marked in a previous study [26]. In our study, replacing water with ethanol-water mixture (60%, *v*/*v*) causes a significant reduction in the extraction rates of lamalbid (**1**), verbascoside (**6**), and quercetin malonylhexoside (**7**), as evidenced by their lower content in the ethanolic-aqueous extract. This in turn might confirm a negative correlation between an increasing percentage of ethanol and total phenols content observed in some plant materials [26]. It is worth noting that compounds quantified in our study represent different classes of compounds characterized by a different physical and chemical nature. The structure of compounds determines their polarity and thus influences their exhaustive recovery. Therefore, structural diversity makes an optimization of extraction challenging [27]. The method developed in this study allowed for simultaneous quantitation of different types of compounds. Additionally, this method is suitable for both aqueous and hydroalcoholic extracts. Nevertheless, the phenolic profile is strongly affected by the selection of the extraction procedure, which should be carefully revised based on different approaches [27]. In addition, iridoid and phenol content changes with plant growth, and particularly phenol content seems to be time-dependent according to the phenological phase [25,26]. Thus, seasonal variations directly influence the chemical quality and, indirectly, the potential biological activity of plant material. Therefore, harvesting time of *L. album* flowers should also be taken into consideration for further investigations on its standardization for routine control in practice.

The results of quantitative determination of lamalbid (**1**), chlorogenic acid (**3**), rutin (**4**), verbascoside (**6**), quercetin malonylhexoside (**7**), and tiliroside (**8**) in the aqueous and ethanolic-aqueous extracts from *Lamium album* flowers are presented in Table 2. The content of tiliroside (**8**) significantly differs from the previously reported (18.1 ± 1.3 µg g^−1^ d.w. of plant material) [28], which can be connected to several factors. Firstly, in the study by Nowak the content of tiliroside was determined with RP-HPLC after solid-phase extraction (SPE) separation [28]. Therefore, the extraction procedure might have seriously influenced its final quantification. Secondly, the discrepancy may be a result of the recalculation of the obtained tiliroside (**8**) content per dry weight of plant material in the study of Nowak [28]. The contents of phenolic acids such as chlorogenic acid (39.3 mg g^−1^), sinapic acid (61.8 mg g^−1^), and rosmarinic acid (40.8 mg g^−1^) were previously established with HPLC–DAD in methanolic extracts of *L. album* herb [22]. In fact, this quantification showed a higher content of chlorogenic acid (**3**) than in our study. Differences between the used solvents probably indicate methanol is more suitable for the extraction of chlorogenic acid (**3**). Our results for chlorogenic acid (**3**) showed a higher content than both in chloroform extract reported by Veleva et al. [22] and in *Lamium* in situ, *Lamium* in vitro, and *Lamium* ex vitro during micropropagation procedures reported by Kapchina-Toteva et al. [23]. As far as other phenolic acids are concerned, we detected neither rosmarinic acid nor sinapic acid in our previous report [17]. Apart from chlorogenic acid (**3**) and tiliroside (**8**), levels of rare phytoecdysteroids evaluated using radioimmunoassay-guided and HPLC–DAD analysis were determined in the different parts of *L. album* previously [29]. However, taking into consideration the singularity and scarcity of ecdysteroids in this plant material, the practical application of this method is limited. Therefore, we decided to develop a method of quantitative determination of iridoids and phenolic compounds which occur widely in natural products and can be easily used for standardization.

Phenylpropanoid glycosides are widely distributed in species belonging to the subfamily of Lamioideae, contrary to Nepetoideae. Despite singular exceptions (e.g., martynoside from the class of phenylpropanoids has been identified in *Salvia officinalis*), it was even concluded that phenylpropanoids might be considered the chemotaxonomic markers differentiating these two subfamilies [30]. In our previous study, we identified few phenylpropanoids including verbascoside (**6**), lamiusides A, B, D, and E in the herb of *L. album* [16,17]. According to previous data phenylpropanoids, such as verbascoside (**6**) and isoverbascoside, may reach up to 55% of the total phenolic content in a SPE-purified ethanolic extract [21]. Phenotypically *L. album* is closely related to *Ballota nigra* L., which is rich in essential oils like many species of Lamiaceae, but the quantitative factor selected for its standardization is verbascoside (acteoside). The content of *o*-dihydroxycinnamic acid expressed as verbascoside (**6**) equivalent should not be less than 1.5% in a dry herb of *B. nigra* by a definition of Ph. Eur. monograph (European Pharmacopoeia 9th Ed.) [7]. In addition, the colorimetric assay is used for the determination of verbascoside (**6**) content in raw plant material. In the study on phenolic composition of *B. nigra* infusion, the concentrations of verbascoside (**6**) and chlorogenic acid (**3**) were established as 10.3 and 1.8 mg g^−1^, respectively [31]. Both phenylpropanoids and phenolic acids/depsides are often used in the standardization of plant materials. Thus, chlorogenic acid (**3**) and verbascoside (**6**) have been particularly proposed as reference compounds for routine quantitative control of *Lamii albi flos*, also due to the commercial availability of chemical standards. Nevertheless, lamalbid (**1**) along with caryoptoside (**2**) are considered characteristic for *L. album* [18]. In our study, lamalbid (**1**) has been the most abundant compound of the studied extracts. In addition, it plays a role in a phylogenetic recognition and differentiation within *Lamium* genus [19], and its biological significance has been preliminary proved [16]. In addition to the interleukin 8 (IL-8) and tumor necrosis factor *α* (TNF-*α*) inhibitory activity in human neutrophils, lamalbid (**1**) has been reported to inhibit reactive oxygen species production and scavenge 2,2-diphenyl-1-picrylhydrazyl radical (66.56%) at a concentration of 100 µg mL^−1^ [16,32]. For this reason, the quantitation of lamalbid (**1**) might be also included in the assessment of *Lamii albi flos* quality.

Presently, the Commision E monograph lists an internal use of preparations from the white dead nettle in treatment of the upper respiratory tract disorders, or as a topical medication for mild inflammation of the throat, mouth, and skin [8,33]. The recently published in vivo results showed that *L. album* extracts reduced the COX-2 expression in livers of diabetic rats, as well as decreased blood glucose levels [11,13]. The present role of *L. album* in the traditional medicine seems to have been neglected, despite the commercial availability of dried herbs used for homemade preparations. In addition, the usage of *L. album* herb in the form of spice or salads enhance its dietary importance in some European regions. Therefore, the standardization of commercially available plant materials seems to be worthy of investigation in order to provide a product of an appropriate quality.

## 3. Materials and Methods

### 3.1. Chemicals

Acetonitrile HiPerSolv Chromanorm^®^ was purchased from VWR Chemicals (Radnor, PA, USA), HPLC grade ethanol and methanol were purchased from POCh (Gliwice, Poland), formic acid (98–100%) was purchased from Merck (Darmstadt, Germany). Water was purified with the Millipore Simplicity System (Bedford, MA, USA). Standard substances: lamalbid (**1**), caryoptoside (**2**), chlorogenic acid (**3**), rutin (**4**), lamiuside A (**5**), verbascoside (**6**), quercetin malonylhexoside (**7**), and tiliroside (**8**) were isolated from *L. album* herb in the Department of Pharmacognosy and Molecular Basis of Phytotherapy, Medical University of Warsaw, Poland. The structures of compounds (UV–Vis and ^1^H spectra), except for chlorogenic acid (**3**) and quercetin malonylhexoside (**7**), were described in our previous research. The molecular masses and the purity of compounds (>95%) were determined using high-performance liquid chromatography coupled with diode detector and mass spectrometer (HPLC–DAD-MS*^n^*) method [16,17]. Lamalbid (**1**) was characterized by an [M + HCOOH − H]^−^ ion at *m/z* 467 in negative ESI mode. The major MS^2^ ion in negative ionization mode was [M-H]¯ *m/z* 421 and [M – H – Glc]^−^ *m/z* 259. The MS data of chlorogenic acid (**3**) showed peaks at *m/z* 353 [M − H]^−^, and fragmentary ion at *m/z* 191. The main ion in the MS spectrum of verbascoside (**6**) was [M − H]^−^ (*m/z* 623) in the negative ESI mode, whereas the main ions in the MS^2^ pattern in negative ESI mode were at *m/z* 461 and *m/z* 315. The quercetin derivatives such as rutin (**4**) and quercetin malonylhexoside (**7**) showed signals at *m/z* 609 [M − H]^−^ and *m/z* 549 [M − H]^−^, respectively. Their MS^2^ fragmentation patterns showed signals at *m/z* 463 and 301 for rutin (**4**) and *m/z* 505, 463 and 301 for quercetin malonylhexoside (**7**) in the negative ionization mode. The MS data of tiliroside (**8**) showed peaks at *m/z* 593 [M − H]^−^, and fragmentary ions at *m/z* 447 and *m/z* 285 [16,17].

### 3.2. Plant Material and Extracts Preparation

*Lamium album* L. flowers were collected in May 2017 in Warsaw (52°12′47″ N, 20°59′52″ E). A specimen (No FW25_20170425_LA) of *L. album* is available in the herbarium of the Department of Pharmacognosy and Molecular Basis of Phytotherapy, Medical University of Warsaw. The plant material was identified according to *Flora Europea* [34].

### 3.3. Extracts Preparation

Aqueous extract: a 19.5-g portion of powdered plant material was extracted three times with boiled water (200 mL) for 15 min each time. The collected aqueous extracts were concentrated under reduced pressure and lyophilized.

Ethanolic-aqueous extract: a 19.5-g portion of powdered plant material was macerated three times with aqueous ethanol (60%, *v*/*v*; 300 mL) in a ratio of 1:15 for 24 h each time at room temperature in the darkness. The collected ethanolic extracts were concentrated under reduced pressure and the aqueous residue was lyophilized afterwards.

The lyophilized residues were powdered using a mortar. The dry weights of the aqueous and ethanolic-aqueous extracts from the flower samples were 4.84 g and 6.57 g, respectively.

### 3.4. Sample Solutions

Accurately weighted 40 mg of extracts’ samples were dissolved in 1 mL of 0.1% (*v*/*v*) formic acid in water. Solutions were filtered through 0.45 µm La-Pha-Pack syringe filters (Langerwehe, Germany) to HPLC vials ND8 Agilent Technologies (Santa Clara, CA, USA).

### 3.5. Standards Solutions

Accurately weighted amounts of standards were dissolved in appropriate volume of 0.1% (*v/v*) formic acid in water to obtain concentrations: 4 mg mL^−1^ for lamalbid (**1**) solution, 2 mg mL^−1^ for chlorogenic acid (**3**) and verbascoside (**6**) solutions, 1 mg mL^−1^ for rutin (**4**) and quercetin malonylhexoside (**7**) solutions, and 0.5 mg mL^−1^ for tiliroside (**8**).

The aliquots of stock solutions were diluted in 0.1% (*v/v*) formic acid in water to obtain standard solutions containing: 700, 1000, 1250, 1500, and 1700 µg mL^−1^ for lamalbid (**1**), 125, 250, 350, 450, 550, and 650 µg mL^−1^ for chlorogenic acid (**3**), 62.5, 125, 250, 500, and 750 µg * mL^−1^ for verbascoside (**6**), 50, 100, 150, 200, and 300 µg mL^−1^ for rutin (**4**), 50, 100, 200, 300, and 400 µg mL^−1^ for quercetin malonylhexoside (**7**), and 10, 30, 50, 80, and 100 µg mL^−1^ for tiliroside (**8**).

### 3.6. Chromatography Analysis

The HPLC–DAD analyses were performed using an apparatus equipped with a dual low-pressure gradient pump LC-10AT, a sampler SIL-20A, a CTO-10AS column oven set at 25 °C, and a diode-array detector SPD-M20A (all, Shimadzu, Kyoto, Japan). HPLC analyses were carried out on a reversed-phase Kinetex XB-C_18_ column (150 × 2.1 mm, 2.6 μm; Phenomenex, Torrance, CA, USA). The mobile phase (A) was 0.1% formic acid in water (*v*/*v*), and the mobile phase (B) was 0.1% formic acid in acetonitrile (*v/v*). A multistep gradient solvent system, 0–17 min, 10%–19% B, 17–37 min, 19%–21% B, and 37–50 min, 21%–40% B was used. The flow rate was 0.2 mL/min. The column was equilibrated using 10% B for 10 min between injections. Volume of injected samples was 2 µL.

UV–Vis spectra were recorded over a range of 200–450 nm, chromatograms were acquired at 240 nm (iridoids), 320 nm (phenylpropanoids, depsides) and 350 nm (flavonoids). LabSolutions system (Shimadzu, Kyoto, Japan) was used for operating procedures and calculation management, and to provide complete quantitative data. For every tested compound calibration curve was obtained by plotting the peak areas versus the amount of standards. The content of compounds in samples was calculated using the regression parameters of the calibration curves.

### 3.7. Method Validation

The method developed was validated according to the International Conference on Harmonisation (ICH) guidelines [4]. The validation of the method was carried out using the ethanolic-aqueous extract.

#### 3.7.1. Specificity

Specificity was tested by comparing retention times, peaks purity, and UV spectra of substances in extracts with reference substances.

#### 3.7.2. Linearity

To evidence linear relationship between compound signals and concentration of compounds determinations for 5 concentrations in minimum triplicate were performed. Correlation coefficient, y-intercept, slope of the regression line, and residual sum of squares were provided (Supplementary Materials, Schemes S1–S6).

#### 3.7.3. Range

The working ranges were defined as ranges between limit of quantification values and the highest compound concentrations from linearity determinations.

#### 3.7.4. Accuracy

The accuracy of the method was assessed using three concentration levels covering the specified range. Accuracy was assessed by testing the recovery of nine sample solutions. It was reported as percent recovery at levels 60, 100, and 140% of the known added amounts of the tested analytes in the sample. A sample solution (40 mg mL^−^^1^) and standard solution in the determined concentration were mixed in the ratio 1:1 to obtain appropriate content of the tested compound. The ratio of known added concentration of standard compound to the concentration of standard compound calculated based on the calibration curve developed with the HPLC–DAD method was determined, and expressed as a percentage of recovery.

#### 3.7.5. Precision

Precision was assessed by testing the repeatability of six independent sample solutions (intraday) and by intermediate precision analysing three independent sample solutions on different days (interday).

#### 3.7.6. Limit of Detection (LOD) and Limit of Quantification (LOQ)

Approaches for determining limit of detection (LOD) and limit of quantification (LOQ) were established based on the standard deviation of the response (*σ*—standard deviation of the intercept) and the slope (*S*) of the calibration curves. Limits of detection and quantitation were calculated using the formulas: LOD = 3.3 × *σ*/*S* and LOQ = 10 × *σ*/*S*, respectively [4].

### 3.8. Statistical

The results, expressed as a mean ± standard deviation (SD) of the indicated compounds, were established by one-way ANOVA. The linearity of calibration curves was confirmed by analysis of residual sum of squares (F test). The data were processed using Excel (Microsoft, USA) and Statistica 13 (StatSoft, Cracow, Poland) software.

## 4. Conclusions

The method developed of separating compounds in aqueous and ethanolic-aqueous extracts from white dead nettle flowers is specific, linear, reproducible, precise, and accurate. The use of a three-stage gradient elution allows for effective separation of all eight tested chemical substances belonging to the different classes of compounds, including iridoids, depsides, flavonoids, and phenylpropanoids. The most abundant compounds of both extracts were lamalbid (**1**), chlorogenic acid (**3**), and verbascoside (**6**). In particular, lamalbid (**1**) seems to be a remarkable chemotaxonomic marker of *L. album*, and might play a role of standard compound for this plant material. However, taking into consideration the pharmacopoeial requirements, as well as the availability of compounds, chlorogenic acid (**3**) and verbascoside (**6**) would be preferentially selected for quantitative assessment of *L. album* flowers. The addition of ethanol results in a decrease in the content of iridoids and phenylpropanoids in the extract. Thus, the use of more popular aqueous preparations of *Lamii albi flos* in traditional medicine seems to be justified and confirmed by the data obtained. Further investigation into harvesting time influence is required to develop standardization procedures for routine control of *L. album* flowers in practice.

## Figures and Tables

**Table 1:** Method validation data for the quantitative determination of the six tested compounds.

Parameters of Validation		Compound											
		Lamalbid (1)		Chlorogenic acid (3)		Rutin (4)		Verbascoside (6)		Quercetin Malonylhexoside (7)		Tiliroside (8)	
**Linearity**	**Regression Equation**	**y = 5.4985 × 10^6^x + 5.2368 × 106**		**y = 1.4796 × 10^7^x − 11,665.0421**		**y = 9082.6404x + 11,626.4171**		**y = 8.0551 × 10^6^x − 45,113.1144**		**y = 6914.3069x − 79,129.6939**		**y = 16,857.3222x + 14,746.2045**	
	r	0.9751		0.9923		0.9964		0.998		0.9958		0.9853	
	R^2^	0.9508		0.9847		0.9928		0.9961		0.9916		0.9709	
	test F (α = 0.99)	444.19		1805.34		211.16		5825.75		2710.48		767.05	
**Recovery (n = 9)**		(%)	CV (%)	(%)	CV (%)	(%)	CV (%)	(%)	CV (%)	(%)	CV (%)	(%)	CV (%)
	60% of content	118.79	1.71	109.88	9.08	103.53	1.42	96.19	2.79	108.03	0.21	108.44	7.08
	100% of content	115.55	4.12	96.49	7.20	96.57	9.25	140.52	9.53	109.02	5.82	90.12	8.92
	140% of content	111.17	2.22	110.51	4.14	98.33	4.93	101.94	2.26	97.48	5.07	97.73	2.24
**Repeatability (n = 6)**	mean (ng)	2117.47		871.99		139.95		474.20		308.65		111.53	
	S (ng)	70.16		115.14		23.46		13.73		13.37		8.97	
	CV (%)	3.30		13.20		16.76		2.90		4.30		8.04	
	$\tilde{x}\;\pm \Delta x$ (α = 0.05) (ng)	2117.47 ± 50.00		871.99 ± 115.02		139.95 ± 23.44		474.20 ± 13.72		308.65 ± 13.36		111.53 ± 8.96	
**Intermediate Precision (n = 6)**	mean (ng)	2131.45		971.62		141.04		474.20		321.50		109.41	
	S (ng)	50.00		133.02		13.52		10.64		16.67		7.89	
	CV (%)	2.35		13.69		9.59		2.24		5.19		7.21	
	$\tilde{x}\;\pm \Delta x$ (α = 0.05) (ng)	2131.45 ± 20.00		971.62 ± 65.87		141.04 ± 6.69		474.20 ± 6.14		321.50 ± 8.25		109.41 ± 7.88	
**LOQ ^1^ (ng) (n = 6)**		1950		264		55		140		214		51	
**LOD ^2^ (ng) (n = 6)**		640		87		18		46		70		17	
**Range (ng)**		1950–3400		264–1300		55–600		140–1500		214–800		51–200	

CV—coefficient of variation; S—standard deviation; ^1^ LOQ—limit of quantification (per injection); ^2^ LOD—limit of detection (per injection).

**Table 2:** Contents of the tested compounds in aqueous and ethanolic-aqueous extracts from *Lamium album* flowers (mg g^−1^ d.w. of extract].

Compound	Aqueous extract (mg g^−1^) (n = 6)	Ethanolic-aqueous extract (mg g^−1^) (n = 6)
**Lamalbid (1)**	39.09 ± 1.02	26.66 ± 0.64
**Chlorogenic acid (3)**	9.09 ± 0.20	12.24 ± 1.40
**Rutin (4)**	2.57 ± 0.12	1.76 ± 0.15
**Verbascoside (6)**	7.80 ± 0.36	5.93 ± 0.13
**Quercetin malonylhexoside (7)**	8.19 ± 1.07	4.02 ± 0.16
**Tiliroside (8)**	0.90 ± 0.09	1.37 ± 0.10

## Supplementary Materials

The following are available online: Figures S1–S6: HPLC–DAD chromatograms of the isolated compounds, Schemes S1–S6: The results of statistical analysis (residual sum of squares).
