# Supplementary material for: Lamalbid, Chlorogenic Acid, and Verbascoside as Tools for Standardization of Lamium album Flowers—Development and Validation of HPLC–DAD Method
Source: Molecules. 2020 Apr 9;25(7):1721. doi: 10.3390/molecules25071721 (PMC7180761; doi:10.3390/molecules25071721)

Supplementray Materials

Lamalbid, chlorogenic acid, and verbascoside as the tools for standardization of *Lamium album* flowers- development and validation of HPLC-DAD method

Monika E. Czerwińska [1,*], Eleonora Kalinowska[2], Dominik Popowski [1], and Agnieszka Bazylko [1]

[1] Department of Pharmacognosy and Molecular Basis of Phytotherapy, Medical University of Warsaw, 1 Banach street, 02-097 Warsaw, Poland; mczerwinska@wum.edu.pl (M.E.C); dominik.popowski@wum.edu.pl (D.P.); agnieszka.bazylko@wum.edu.pl (A.B.)

[2] Students' Scientific Association at the Department of Pharmacognosy and Molecular Basis of Phytotherapy, Medical University of Warsaw, 1 Banach street, 02-097 Warsaw, Poland; eleonora_kalinowska@op.pl

* Correspondence: mczerwinska@wum.edu.pl; Tel+48-22-572-0953

Lamalbid (1)

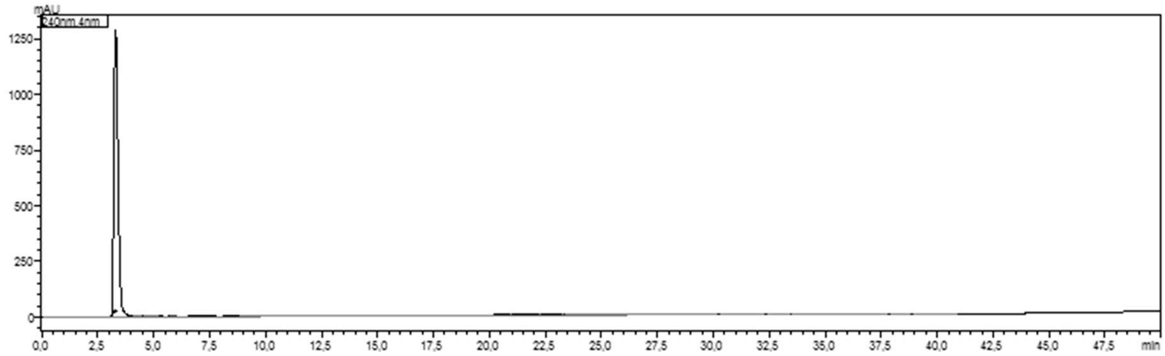

Figure S1. HPLC-DAD chromatogram registered for standard of lamabid (1).

Scheme S1. Analysis of residual sum of squares for lamalbid (1).

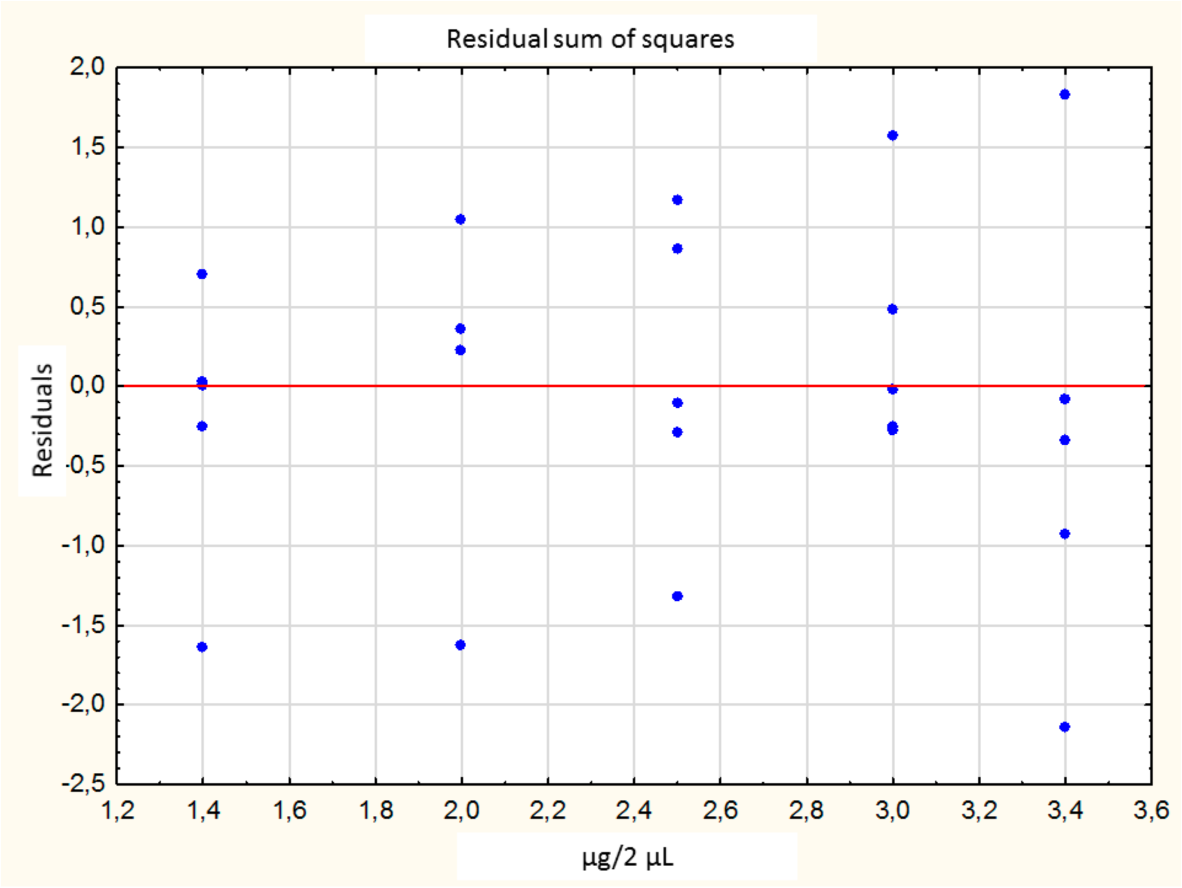

Chlorogenic acid (3)

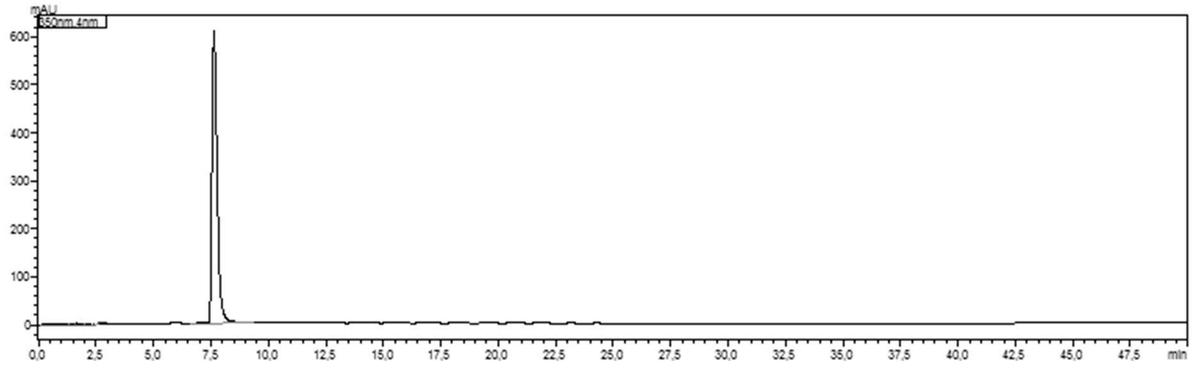

Figure S2. HPLC-DAD chromatogram registered for standard of chlorogenic acid (3).

Scheme S2. Analysis of residual sum of squares for chlorogenic acid (3).

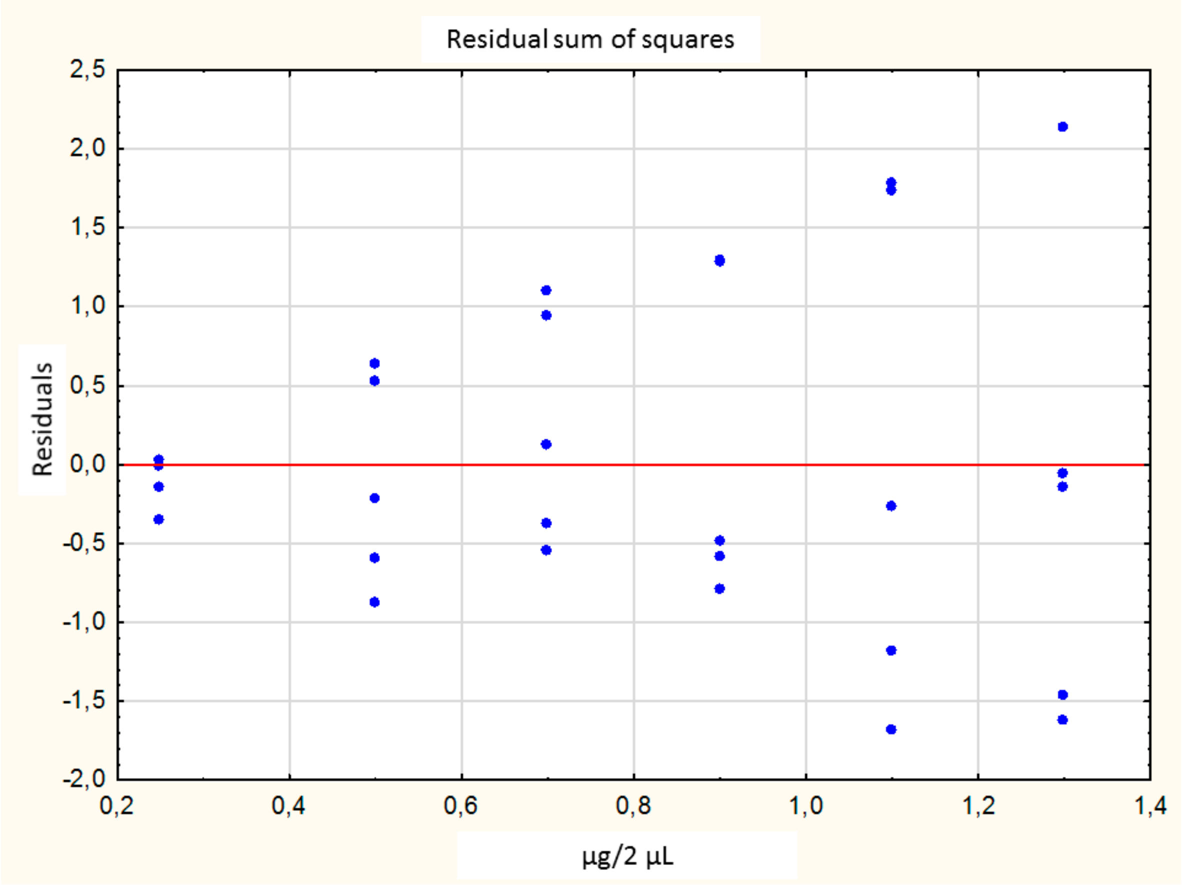

Rutin (4)

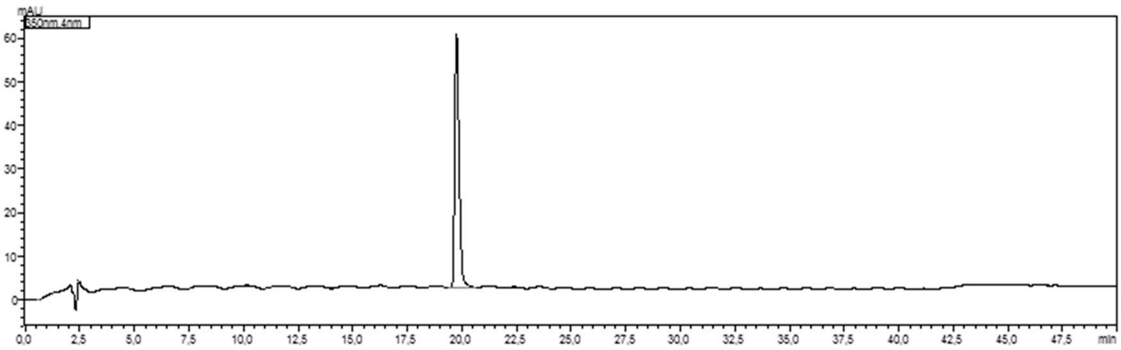

Figure S3. HPLC-DAD chromatogram registered for standard of rutin (4).

Scheme S3. Analysis of residual sum of squares for rutin (4).

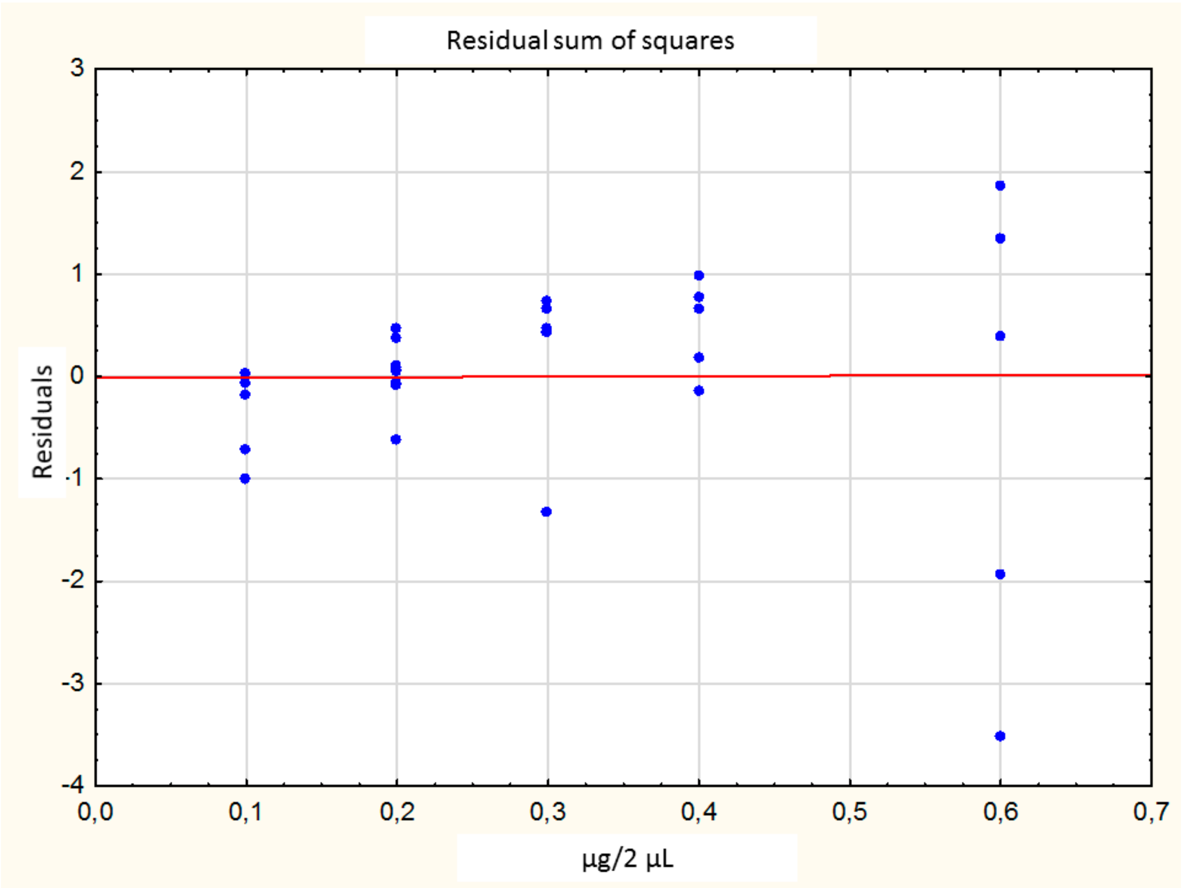

Verbascoside (6)

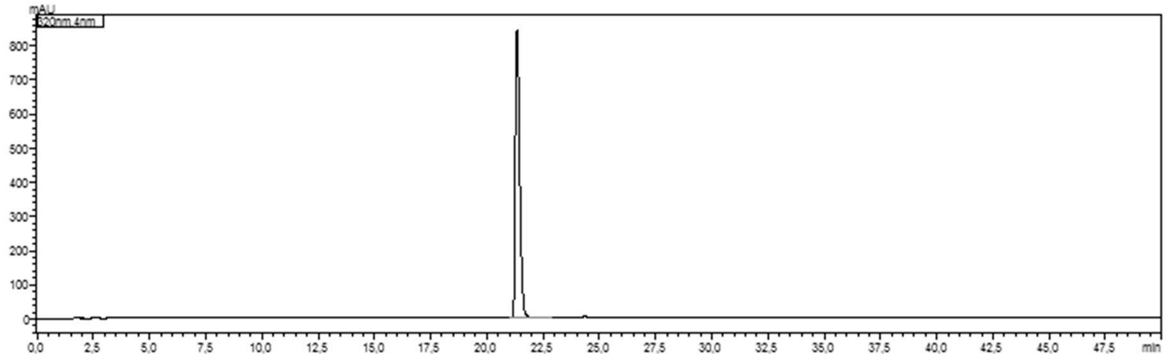

Figure S4. HPLC-DAD chromatogram registered for standard of verbascoside (6).

Scheme S4. Analysis of residual sum of square for verbascoside (6).

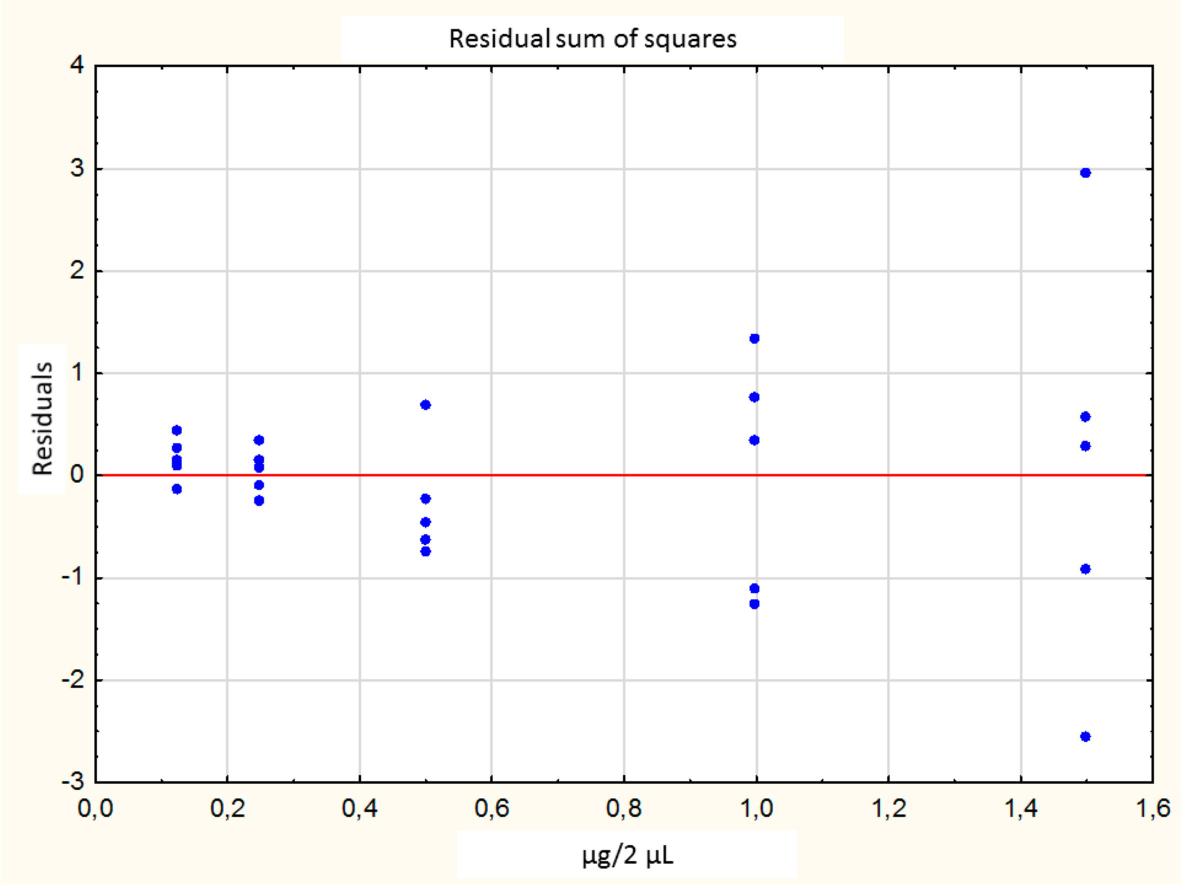

Quercetin malonylhexoside (7)

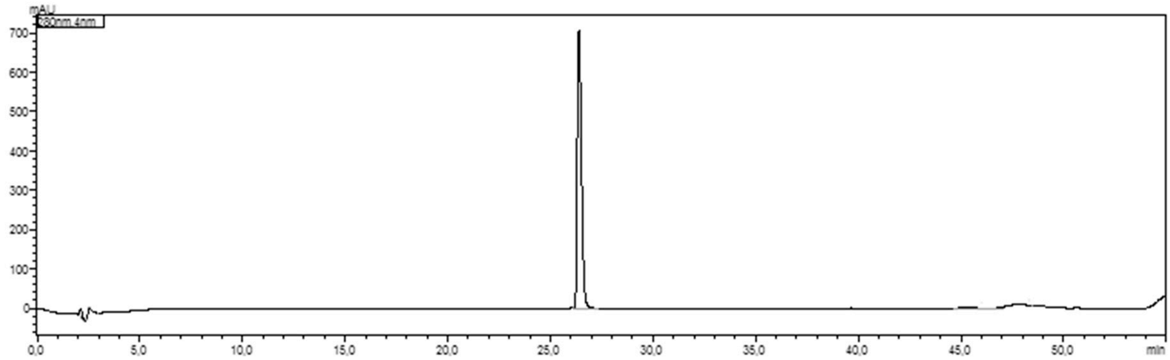

Figure S5. HPLC-DAD chromatogram registered for standard of quercetin malonylhexoside (7).

Scheme S5. Analysis of residual sum of squares for quercetin malonylhexoside (7).

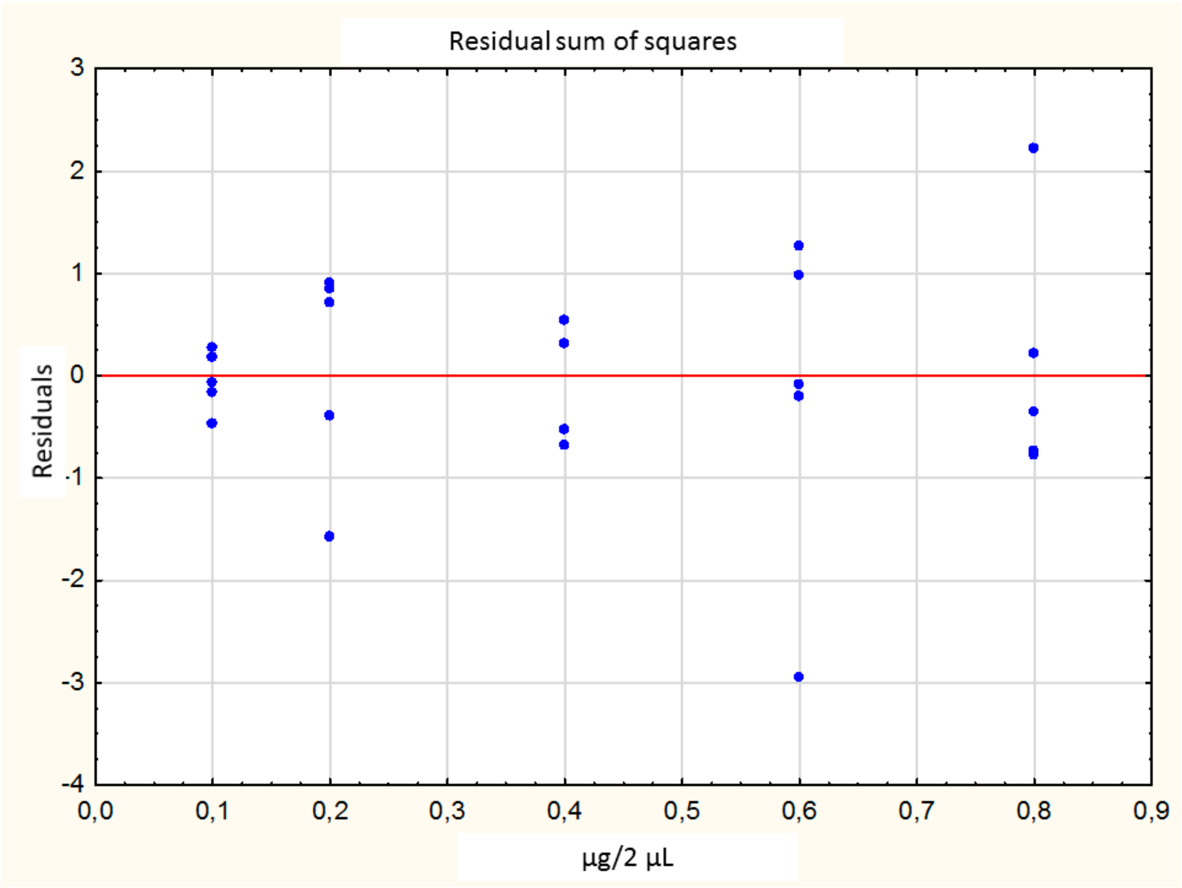

Tiliroside (8)

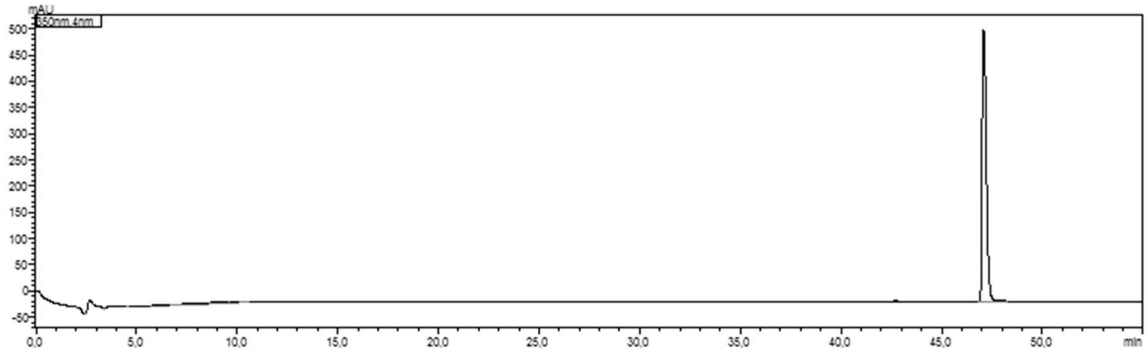

Figure S6. HPLC-DAD chromatogram registered for standard of tiliroside (8).

Scheme S6. Analysis of residual sum of squares for tiliroside (8).

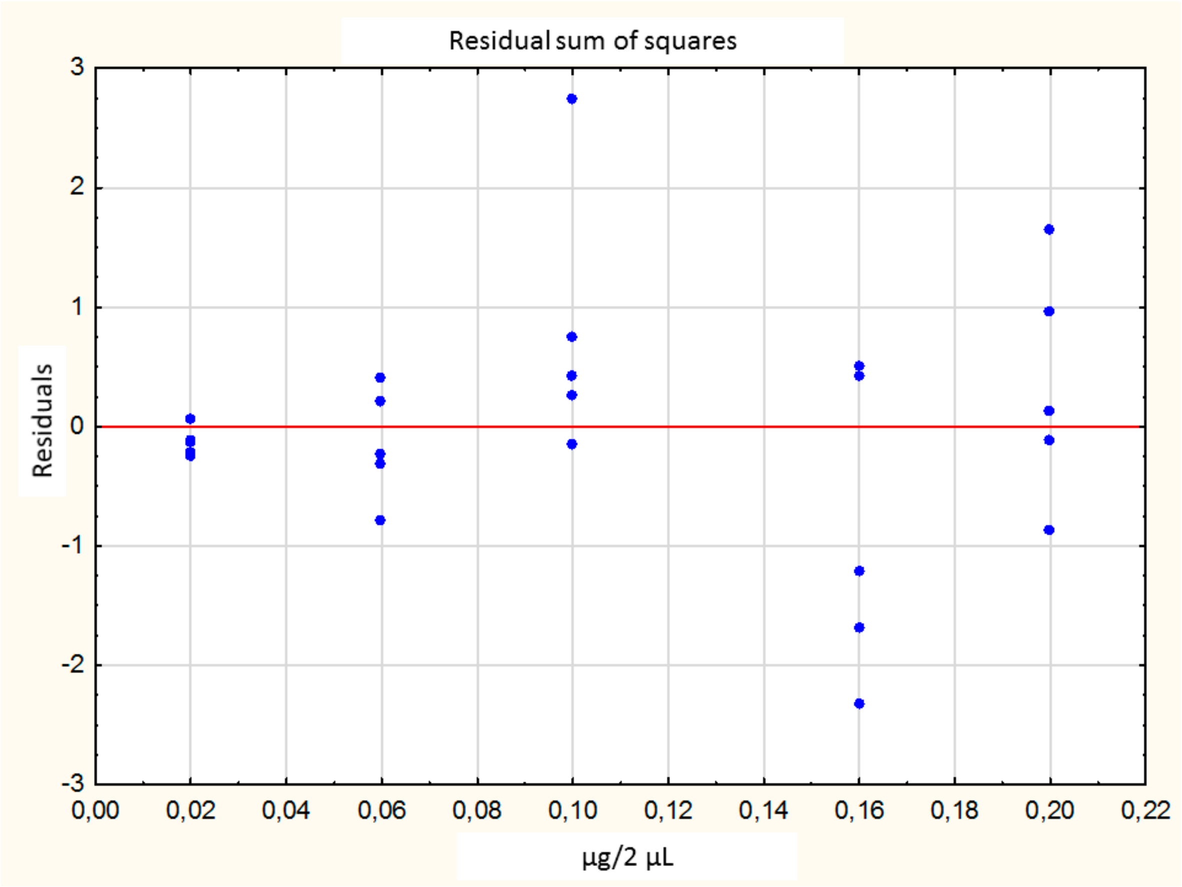

Supplement: Supplementary file 1 [file molecules-25-01721-s001.pdf]
